# COL6A2 drives clear cell renal cell carcinoma progression via integrin-dependent modulation of Wnt/β-catenin signaling

**DOI:** 10.7150/jca.120607

**Published:** 2025-11-14

**Authors:** Xing Ji, Yongyang Yun, Zhenpeng Zhu, Tianyu Wu, Mingjian Ruan, Yu Fan, Qian Zhang

**Affiliations:** 1 Department of Urology, Beijing Shijitan Hospital, Capital Medical University, Beijing 100038, China.; 2 Department of Urology, Peking University First Hospital, Beijing, 100034, China.; 3 Institution of Urology, Peking University, Beijing, 100034, China.; 4 National Urological Cancer Center, Beijing, 100034, China.; 5 Beijing Key Laboratory of Urogenital Diseases (Male) Molecular Diagnosis and Treatment Center, Beijing, 100034, China.

**Keywords:** COL6A2, Clear Cell Renal Cell Carcinoma, Wnt/β-catenin signaling pathway, Integrin, Epithelial-Mesenchymal Transition, Prognosis

## Abstract

**Introduction:** The mechanistic role of COL6A2, an extracellular matrix protein, in clear cell renal cell carcinoma (ccRCC) is largely unexplored. This study aimed to investigate COL6A2 expression, its prognostic value, biological functions, and underlying molecular mechanisms in ccRCC.

**Methods:** COL6A2 expression was analyzed in ccRCC tissues and cell lines using public datasets and Western blotting on clinical samples and cell lines. Prognostic associations were evaluated using TCGA-KIRC data via clinicopathological correlations, Kaplan-Meier survival, and Cox regression analyses. Functional effects of COL6A2 knockdown in ccRCC cells were assessed by CCK-8, wound healing, Transwell, and Western blot analysis of EMT-associated proteins. Mechanistic investigations involved bioinformatic analysis, co-immunoprecipitation, Western blotting for Wnt/β-catenin pathway proteins, integrin blockade, and rescue experiments with the Wnt/β-catenin activator.

**Results:** COL6A2 mRNA and protein were significantly upregulated in ccRCC tissues and cell lines. High COL6A2 expression correlated with aggressive clinicopathological features and independently predicted poorer prognosis. COL6A2 knockdown significantly inhibited ccRCC cell proliferation, migration, invasion, and reversed epithelial-mesenchymal transition (EMT). Mechanistically, COL6A2 was found to physically interact with integrin β1, thereby activating the Wnt/β-catenin signaling pathway to induce EMT. Rescue experiments confirmed the role of this signaling axis in mediating the malignant phenotypes.

**Conclusion:** COL6A2 promotes ccRCC aggressiveness and modulates Wnt/β-catenin signaling in an integrin-dependent manner. These findings nominate the COL6A2-integrin interface as a potential therapeutic and biomarker axis in ccRCC.

## 1. Introduction

As the most common histological variant of renal cell carcinoma (RCC), clear cell RCC (ccRCC) constitutes approximately 70% of all diagnoses. Collectively, RCC is a leading malignancy of the genitourinary tract and presents a substantial public health challenge worldwide 1. The widespread adoption of imaging techniques has driven a steady increase in the global incidence of renal cancer, with more than 430,000 new diagnoses in 2020 globally 2-7. Surgical resection often yields excellent long-term outcomes for individuals with localized renal cell carcinoma. However, the treatment of advanced or metastatic RCC remains challenging. Current clinical guidelines recommend combination therapies incorporating immune checkpoint inhibitors (ICIs) as the standard first-line therapy, and these strategies have begun to reduce RCC mortality rates 8, 9. Even so, approximately 180,000 deaths still occurred in 2020, and the 5-year overall survival rate for individuals with metastatic RCC remains below approximately 12 % 10, 11. While ICIs have revolutionized the treatment of advanced RCC, disease progression, often driven by primary or acquired resistance, alongside fatal immune-related adverse events (irAEs), persist as the primary drivers of mortality 12-15. Consequently, elucidating the molecular mechanisms of ccRCC and discovering novel biomarkers are imperative for improving therapeutic efficacy and patient outcomes.

Collagen VI (COL6), an extracellular matrix (ECM) protein composed of three distinct α chains (α1, α2, α3). Although traditionally viewed as a structural scaffold, accumulating evidence indicates that COL6 actively involved in the tumor microenvironment (TME) 16-18. COL6A2, which encodes the α2 chain of COL6, has been found to be highly expressed in multiple cancers, including glioma, breast cancer, lung cancer, colorectal cancer, and gastric cancer 19-27. Through regulating pathways such as EGFR, MAPK/ERK, and PI3K/AKT, COL6A2 contributes to epithelial-mesenchymal transition (EMT), drug resistance, and immune evasion, thereby promoting tumor invasiveness and correlating with poor prognosis. In ccRCC, limited studies have associated high COL6A2 expression with adverse patient outcomes; however, its mechanistic role remains unexplored 28.

The present research was designed to determine the impact of COL6A2 on key malignant phenotypes of ccRCC—including proliferation, invasion, and metastasis—while simultaneously delineating the mechanistic basis for its activity.

## 2. Methods

### 2.1. Bioinformatic Analysis

Using publicly available datasets, we retrieved transcriptional profiles and corresponding clinical data for ccRCC from the TCGA-KIRC project (https://portal.gdc.cancer.gov/projects/TCGA-KIRC) and the Gene Expression Omnibus (https://www.ncbi.nlm.nih.gov/geo/). The TCGA-KIRC dataset provided gene expression data and clinical details for 539 ccRCC tumor samples and 72 adjacent normal tissue samples. Additionally, GEO datasets GSE167093 and GSE40435 provided 254 and 101 paired ccRCC and adjacent normal tissue samples, respectively. Differential expression analysis and survival analysis were performed in R (v4.3.1) with the 'stats', 'car', and 'survival' packages, and the 'ggplot2' package for all data visualization.

To explore the potential oncogenic role of COL6A2 in ccRCC, enrichment analyses were conducted. Differential gene expression analysis was conducted on the TCGA-KIRC dataset to compare tumors with high and low COL6A2 expression, utilizing the 'DESeq2' package in R. to interpret the biological significance of these differentially expressed genes (DEGs), we then conducted pathway and gene set enrichment analyses. For pathway-level analysis (Gene Ontology and KEGG), we used the 'clusterProfiler' and 'GOplot' packages with a filtered list of DEGs (|LogFC| > 1.5, adjusted p.adj < 0.05). For a broader functional assessment, Gene Set Enrichment Analysis (GSEA) was performed on all DEGs meeting the significance threshold of adjusted p.adj < 0.05 29. GSEA was performed using the 'clusterProfiler' package with the Hallmarks gene set (h.all.v7.5.1.symbols.gmt), applying thresholds of FDR (q-value) < 0.25 and p.adjust < 0.05 to identify COL6A2-related pathways in ccRCC. All enrichment results were visualized using the 'ggplot2' package.

To evaluate potential interactions between COL6A2 and cell-surface receptors at the transcript level, we analyzed co-expression in the TCGA-KIRC cohort. Correlations between COL6A2 and the collagen-binding integrin subunits (ITGA1, ITGA2, ITGA10, ITGA11, ITGB1) were computed and summarized as a correlation matrix 30, 31. Heatmaps and gene-gene scatter plots were generated in R using the 'ggplot2' package.

### 2.2. Patient Specimens

Paired ccRCC and adjacent non-tumorous tissue samples were procured from 10 patients who underwent nephrectomy between 2022 and 2023 at the Department of Urology, Peking University First Hospital. Immediately after excision, specimens were snap-frozen and subsequently archived in liquid nitrogen pending downstream experiments. The histological diagnosis of all cases was independently confirmed by at least two certified pathologists at our institution and classified according to the latest WHO Classification guidelines. The protocol for this study was granted ethical clearance by The Ethics Committee of Peking University First Hospital (2024Yan658-001), and all procedures were performed in adherence to the tenets of the Declaration of Helsinki. Written informed consent was obtained from all participants before the study.

### 2.3. Cell Culture, Transfection, and Pharmacological Treatments

All cell lines used in this research were obtained from the American Type Culture Collection (ATCC, USA), including three ccRCC lines (786-O, Caki-1, OSRC-2) and two normal renal lines (HK-2, HEK-293). We selected 786-O and OSRC-2 as primary ccRCC models for functional assays, as both exhibited higher endogenous COL6A2 expression than Caki-1 in our screening (OSRC-2 was also used for co-immunoprecipitation), and included Caki-1 to broaden generalizability across a distinct ccRCC background. HK-2 and HEK-293 (human embryonic kidney) were included as non-malignant kidney-derived comparators to contextualize tumor-associated upregulation and pathway readouts. The standard culture environment consisted of DMEM (Gibco, USA) supplemented with 10% FBS (Invitrogen, USA) and 1% penicillin-streptomycin, in a humidified 5% CO₂ incubator at 37 °C.

Knockdown of COL6A2 was achieved with small-interfering RNA (siRNA). The siRNA sequences used were: si-COL6A2-1 (5′-GGGCCUCCUUCAUCAAGAATT-3′), si-COL6A2-2 (5′-GCAGGCCUGGAUUCAGCUATT-3′), and a non-targeting negative control siRNA (si-NC; 5′-CCUACGCCACCAAUUUCGU-3′). All siRNAs were purchased from Tianyi Huiyuan Co. (Beijing, China). Transfection was performed using Lipofectamine RNAiMAX (Thermo Fisher Scientific, USA) according to the manufacturer's protocol. Briefly, 30 pmol of siRNA and 9 µL of Lipofectamine RNAiMAX were each diluted in 150 µL of Opti-MEM Reduced Serum Medium (Gibco). The diluted solutions were then gently mixed together and incubated for 5 minutes at room temperature. The siRNA-lipid complex mixture was subsequently added dropwise to the cell culture dish. After 60 h, knock-down efficiency was assessed by Western blotting.

For pharmacological experiments, cells were treated with the integrin inhibitor TC-I-15 (HY-107588, MedChemExpress) and/or the Wnt/β-catenin pathway activator CHIR99021 (HY-10182, MedChemExpress). In pathway rescue experiments, COL6A2-silenced cells were treated with 10 µM CHIR99021 for 24 hours. For integrin blockade experiments, cells were treated with 5 µM TC-I-15 for 24 hours. For the combined treatment, cells were co-incubated with 10 µM CHIR99021 and 5 µM TC-I-15 for 24 hours. These treatments were applied before cells were harvested for Western blot or functional analyses.

### 2.4. Western Blotting

Total protein was extracted from cultured cells and tissue specimens using lysis buffer supplemented with protease inhibitors, phosphatase inhibitors, and PMSF. To obtain separate nuclear and cytoplasmic protein lysates, cultured cells were subjected to subcellular fractionation. This was accomplished using a Nuclear and Cytoplasmic Protein Extraction Kit (Beyotime, P0027) as per the manufacturer's protocol. After determining protein concentrations with the BCA assay, samples were prepared for Western blot by separating 20 µg of protein on an SDS-PAGE gel and transferring to a PVDF membrane. The membranes were then blocked in 5% non-fat milk before undergoing sequential incubation with primary and then secondary antibodies. Signals were detected using Pierce™ ECL chemiluminescent substrate, with images captured on a chemiluminescence imaging system (Bio-Rad, USA). Primary antibodies used included anti-COL6A2 (ab180855; Abcam), anti-GAPDH (Proteintech, 10494-1-AP), anti-Histone H3 (Proteintech, 17168-1-AP), anti-Ki-67 (Proteintech, 27309-1-AP), anti-N-cadherin (Proteintech, 22018-1-AP), anti-E-cadherin (Proteintech, 20874-1-AP), anti-Vimentin (Proteintech, 10366-1-AP), anti-β-Catenin (CST, 8480), anti-c-Myc (CST, 5605).

### 2.5. Cell Counting Kit-8 (CCK-8) Assay

After transfection, 786-O and OSRC-2 cells were seeded in 96-well plates (750 cells/well in 100 µL medium). For the next 96 hours, with measurements taken at 24-hour intervals starting from time zero, cell viability was assessed by incubating the cells with 10% CCK-8 reagent (Dojindo, Japan). After maintaining them at 37 °C for 2 hours, the resulting absorbance was recorded at 450 nm.

### 2.6. Wound Healing Assay

Following siRNA transfection, confluent monolayers (> 90%) of 786-O and OSRC-2 cells were first established in 6-well plates. A mechanical scratch was then introduced using a sterile 200 μL pipette tip, and cellular debris was removed with a Phosphate-Buffered Saline (PBS) wash before the addition of fresh serum-free medium. Scratch closure was imaged under an inverted microscope (20 ×, Olympus, Japan) at specific time points (0 h and 12 h) to monitor cell migration. ImageJ software (Fiji v2.14.0/1.54f; NIH, MD, USA) was used to quantify wound closure.

### 2.7. Transwell Assay

After siRNA transfection, the migratory and invasive capacities of 786-O and OSRC-2 cells were evaluated using Transwell chambers (Corning, USA). Migration assays were conducted with uncoated Transwell chambers, whereas invasion assays utilized chambers precoated with Matrigel in the upper compartment. After resuspension in serum-free DMEM, 786-O and OSRC-2 cells were seeded into the upper Transwell chambers at densities of 10,000 and 80,000 cells per well, respectively. Cell migration toward the lower chamber (600 µL of DMEM with 10% FBS) was allowed to proceed for 24 hours at 37 °C. The inserts were then processed for visualization: while the cells on the top side were gently scraped off with a cotton swab, the cells that had successfully moved to the bottom surface were fixed in 4% paraformaldehyde before being stained with 0.1% crystal violet solution. Finally, stained cells were visualized and imaged under an inverted microscope (20 ×, Olympus, Japan). The number of stained cells was quantified using the “Cyto3” model and “livecell_cp3” model within the Cellpose software (v3.1.1.1, GUI/CLI, Python 3.11.10), graphs and statistical analyses were performed in GraphPad Prism 10 (v10.1.0 (264), GraphPad Software, CA, USA) 32.

### 2.8. Co-Immunoprecipitation (Co-IP)

OSRC-2 cells, which exhibit high endogenous COL6A2 expression, were used for Co-IP assays. Cells were lysed with IP lysis buffer (Beyotime, P0013). The cell lysates were centrifuged, and the supernatant was pre-cleared with Protein A/G Magnetic Beads (Beyotime, P2108). Subsequently, the lysates were incubated overnight at 4 °C with anti-COL6A2 antibody (Abcam, ab180855), anti-Integrin β1 antibody (Proteintech, 12594-1-AP), or a normal Rabbit IgG control antibody (Proteintech, 30000-0-AP). The antibody-protein complexes were then captured by adding fresh Protein A/G Magnetic Beads (Beyotime, P2108) and incubating for an additional 4 hours. After washing the beads three times with IP lysis buffer, the immunoprecipitated proteins were eluted by boiling in SDS-PAGE Loading Buffer and analyzed by Western blotting.

### 2.9. Statistical Analysis

Data were statistically analyzed using GraphPad Prism 10 (v10.1.0 (264)) and R (v4.3.1; R Foundation for Statistical Computing, Austria). Continuous variables were compared across groups using appropriate tests such as Student's t-test, one-way ANOVA, Wilcoxon rank-sum test, or Kruskal-Wallis test, depending on the groups' number and data distribution characteristics. Categorical data were compared between groups utilizing either the Chi-square test or Fisher's exact test as appropriate. Logistic regression modeling was used to determine if COL6A2 expression could predict clinicopathological outcomes. For survival comparisons between high- and low-COL6A2 expression groups, both Kaplan-Meier analysis and Cox proportional hazards regression were employed. The identification of prognostic factors involved a multi-step Cox regression approach: variables were first tested in a univariate model, and only those with a p value below 0.1 were advanced for inclusion in a multivariate model. A two-tailed p-value below 0.05 was considered to indicate statistical significance across all analyses.

## 3. Results

### 3.1. COL6A2 is Upregulated in ccRCC

**Figure 1:**
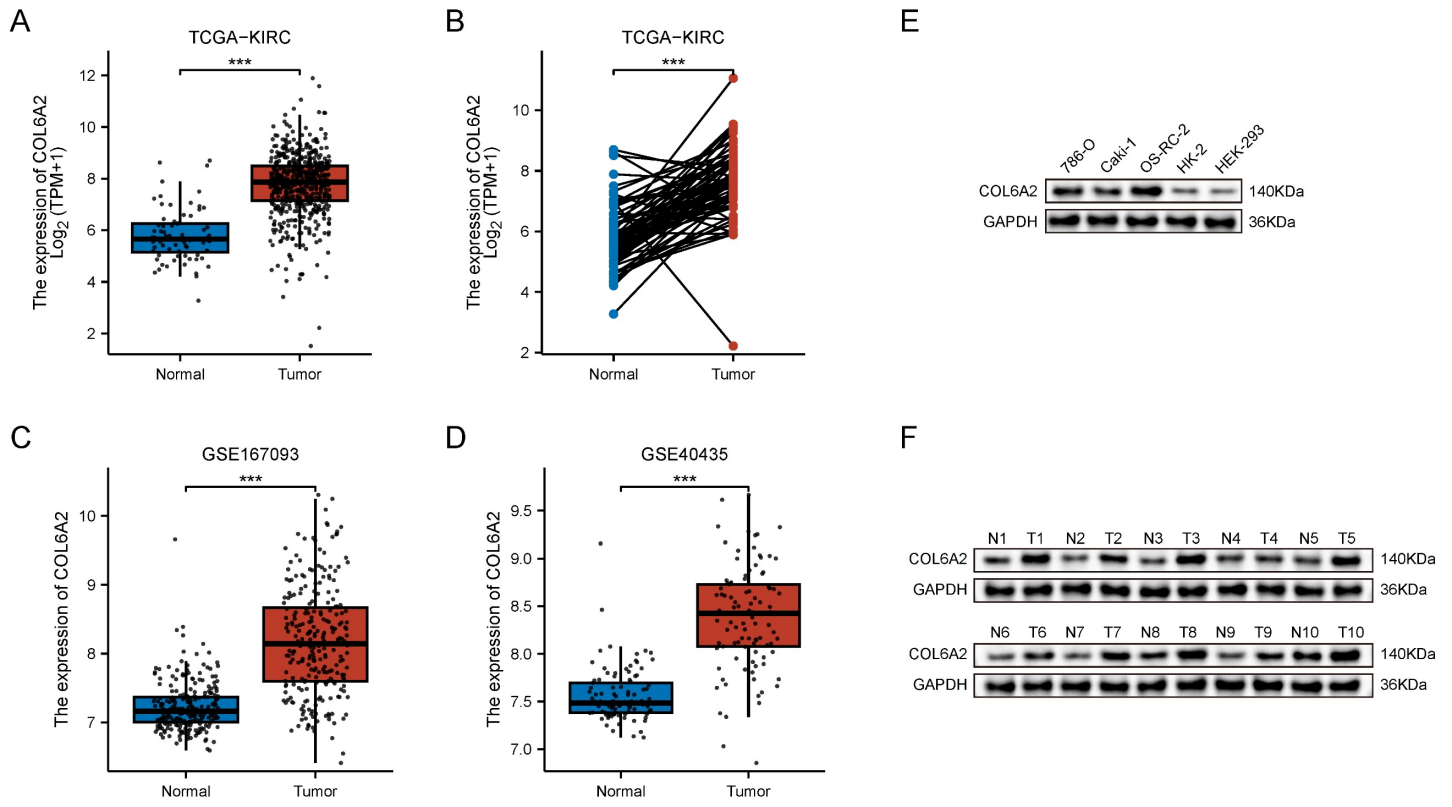
COL6A2 expression in ccRCC. (**A**) Differential COL6A2 mRNA expression between ccRCC and normal kidney tissues (TCGA). (**B-D**) COL6A2 mRNA levels in paired ccRCC and adjacent normal tissues from TCGA (B), GSE167093 (C), and GSE40435 (D). (**E**) COL6A2 expression in ccRCC cell lines and normal renal cell lines. (**F**) COL6A2 protein levels in ccRCC tumor (T) versus adjacent normal (N) tissues. *p < 0.05, **p < 0.01, ***p < 0.001; ns, not significant.

Analysis of the TCGA-KIRC cohort revealed differential COL6A2 transcript expression both in an unpaired comparison of 539 ccRCC tumors against 72 normal tissues and in a paired analysis of 72 matched tumor and adjacent normal specimens. In both unpaired and paired comparisons, COL6A2 mRNA was significantly higher in tumors (Fig. 1A, B). The result was independently validated in two GEO datasets—GSE167093 (254 paired samples) and GSE40435 (101 paired samples)—where tumour tissues likewise displayed significant COL6A2 up-regulation (Fig. 1C, D). Thus, three independent public datasets consistently demonstrate elevated COL6A2 expression in ccRCC.

Protein analysis reached the same conclusion. Western blotting further confirmed a marked upregulation of COL6A2 protein in the ccRCC cell lines (786-O, Caki-1, OSRC-2) relative to the normal renal cell lines (HK-2 and HEK-293) (Fig. 1E). In 10 clinical ccRCC samples from our institution, COL6A2 protein level was consistently higher in tumor tissues compared to the corresponding adjacent non-neoplastic tissue (Fig. 1F). Collectively, these findings demonstrate that COL6A2 is overexpressed in ccRCC at both the transcript and protein levels.

### 3.2. High COL6A2 Expression Correlates with Poor Prognosis in ccRCC

**Figure 2:**
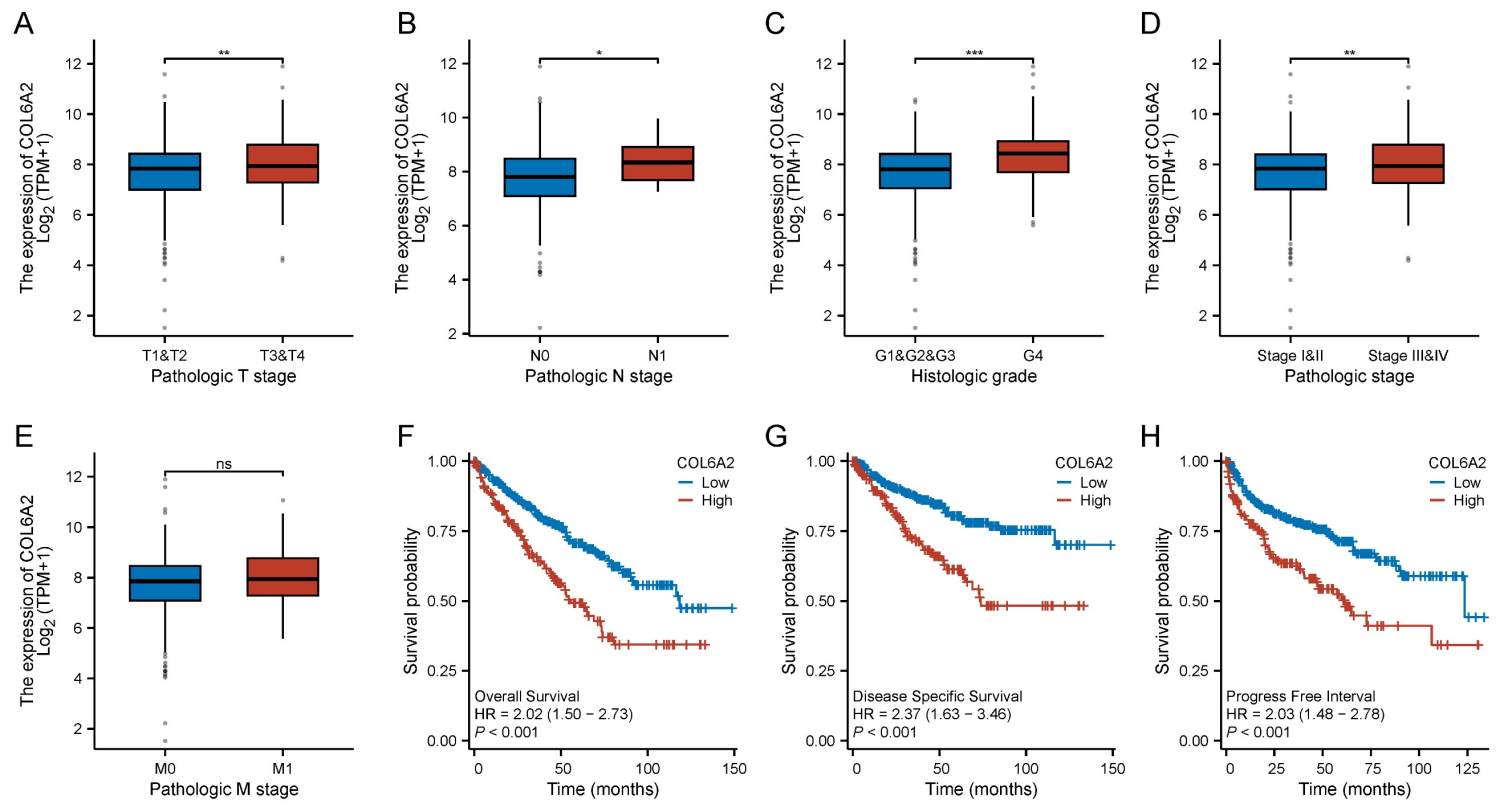
Clinical and prognostic significance of COL6A2 expression in ccRCC (TCGA). (**A-E**) Correlation of COL6A2 mRNA expression with (A) T stage, (B) N stage, (C) histologic grade, (D) pathologic stage, and (E) M stage. (**F-H**) Kaplan-Meier analysis for (F) Overall Survival (OS), (G) Disease-Specific Survival (DSS), and (H) Progression-Free Interval (PFI) based on COL6A2 mRNA expression levels. *p < 0.05, **p < 0.01, ***p < 0.001; ns, not significant.

Analyzing 539 ccRCC patients from the TCGA-KIRC cohort, we found that elevated COL6A2 expression correlates with adverse prognosis. Specifically, high COL6A2 levels were significantly associated with advanced T stage, N stage, poorer histological grade, and later pathological stage (Fig. 2A-D). Patients with distant metastasis trended higher in COL6A2 expression compared to those without metastasis. Although the difference in median expression between the M0 and M1 groups was not statistically significant in a direct comparison (Fig. 2E), logistic regression analysis, which models the odds based on continuous expression data, revealed that increasing COL6A2 expression is a significant predictor for a higher likelihood of distant metastasis (OR = 1.257, P = 0.035) (Table 1).

To further quantify the association between COL6A2 expression and these clinicopathological features, we performed logistic regression analyses. These analyses revealed that higher COL6A2 expression was significantly associated with an increased likelihood of higher T stage (OR = 2.901), lymph node metastasis (OR = 1.574), distant metastasis (OR = 1.257), later pathological stage (OR = 1.294), and advanced histological grade (OR = 1.838) (Table 1). Our findings indicate that high levels of COL6A2 expression in ccRCC are closely associated with a more aggressive clinicopathological characteristics.

Kaplan-Meier analysis demonstrated that high COL6A2 expression was associated with significantly reduced overall survival (OS; HR = 2.02, 95% CI: 1.50-2.73), disease-specific survival (DSS; HR = 2.37, 95% CI: 1.63-3.46), and progression-free interval (PFI; HR = 2.03, 95% CI: 1.48-2.78) (Fig. 2F-H).

To identify independent prognostic factors, we conducted Cox regression analysis incorporating COL6A2 expression and clinical variables such as age, gender, T stage, N stage, M stage, histological grade, and pathological stage. Univariate analysis indicated that high COL6A2 expression (HR = 1.293), older age, advanced T stage, nodal and distant metastasis, higher grade, and later pathologic stage were each linked to worse OS (Table 2). Multivariate analysis confirmed that high COL6A2 expression (HR = 1.354, 95% CI: 1.108-1.656), older age (HR = 1.810, 95% CI: 1.173-2.793), distant metastasis (HR = 2.410, 95% CI: 1.412-4.111), and higher histological grade (HR = 1.663, 95% CI: 1.014-2.729) remained independent predictors of poorer OS in ccRCC (Table 2). Together, these findings establish COL6A2 as a robust marker of aggressive biology and poor outcome in ccRCC.

### 3.3. COL6A2 Knockdown Inhibits Proliferation, Migration, Invasion, and EMT in ccRCC Cells

**Figure 3:**
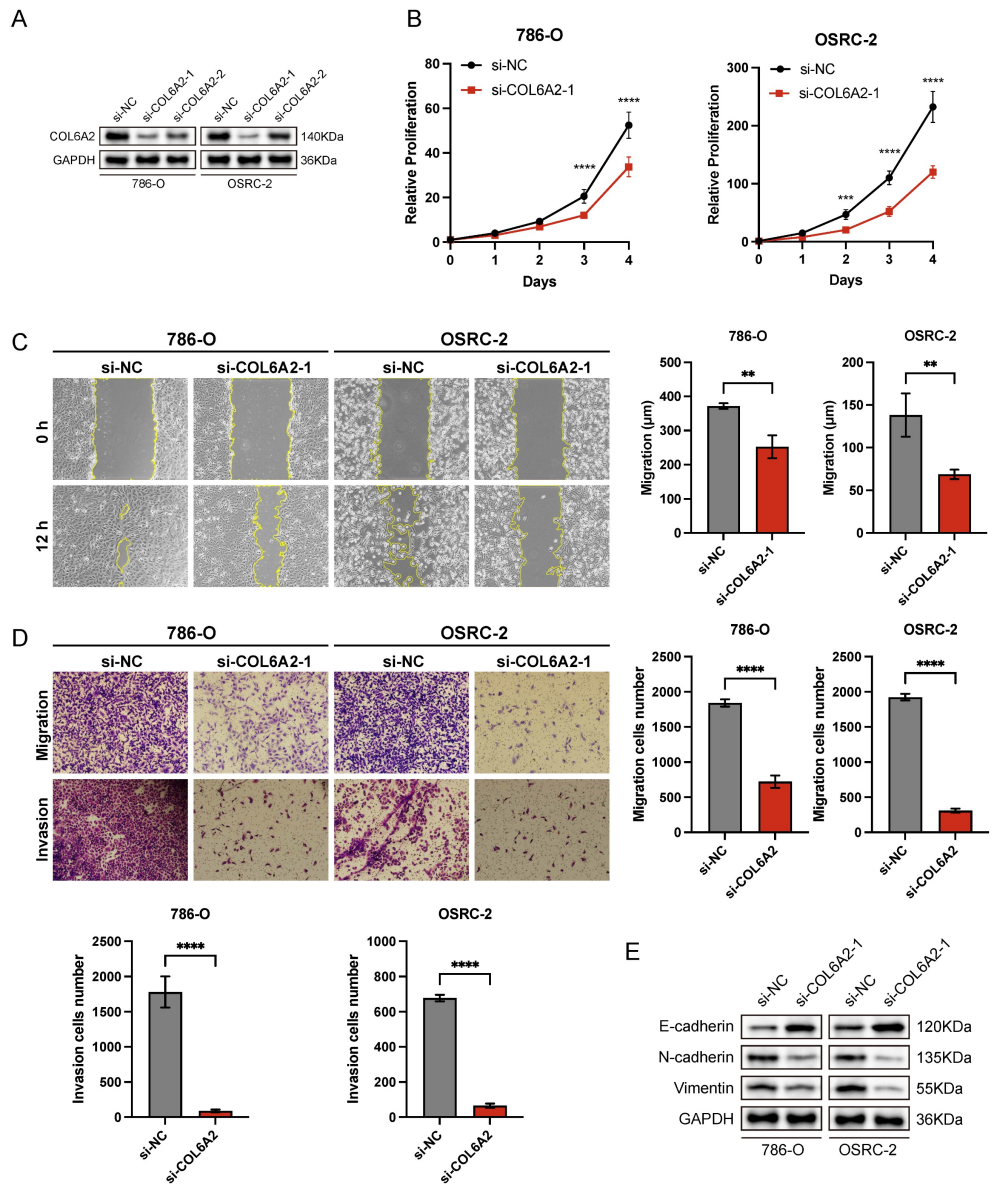
COL6A2 knockdown inhibits proliferation, migration, invasion, and EMT in ccRCC cells. (**A**) Western blot validation of COL6A2 knockdown efficiency in 786-O and OSRC-2 cells. (**B**) Viability of 786-O and OSRC-2 cells following COL6A2 knockdown, assessed by CCK8 assay. (**C**) Wound healing assays revealed COL6A2 knockdown inhibited migration of 786-O and OSRC-2 cells. (**D**) Transwell assays demonstrated COL6A2 knockdown reduced migration and invasion of 786-O and OSRC-2 cells. (**E**) Western blot analysis of EMT-associated protein expression in 786-O and OSRC-2 cells following COL6A2 knockdown. *p < 0.05, **p < 0.01, ***p < 0.001, ****p < 0.0001; ns, not significant.

To explore the biological role of COL6A2 in ccRCC, we selected 786-O and OSRC-2 cell lines, which exhibit elevated COL6A2 expression, for *in vitro* functional studies. COL6A2 was silenced using siRNA, and knockdown efficiency was validated by Western blotting, with si-COL6A2-1 demonstrating superior efficacy (Fig. 3A).

Silencing of COL6A2 expression led to a significant suppression of cell proliferation in both the 786-O and OSRC-2 cell lines. This effect was quantified using a CCK-8 assay, which showed a substantially lower growth rate in COL6A2-depleted cells compared to those transfected with a negative control (si-NC) (Fig. 3B). These results suggest that COL6A2 silencing markedly suppresses ccRCC cells proliferation.

The role of COL6A2 in mediating the motility of ccRCC cells was further investigated through a panel of functional assays, including a wound healing assay for migration, as well as Transwell assays to quantify both cell migration and invasion. The results revealed that COL6A2 knockdown significantly suppressed the migratory and invasive abilities of both 786-O and OSRC-2 cells (Fig. 3C, D), indicating a pivotal role for COL6A2 in regulating these processes. To investigate the role of COL6A2 in epithelial-mesenchymal transition (EMT), we examined key marker proteins following its knockdown. The results indicated a significant upregulation of the epithelial marker E-cadherin, which occurred in parallel with a marked decrease in the expression of the mesenchymal markers N-cadherin and vimentin (Fig. 3E). These findings suggest that COL6A2 silencing reverses EMT, promoting an epithelial phenotype in ccRCC cells.

In summary, COL6A2 is critical for ccRCC cell proliferation, migration, and invasion, and it may facilitate ccRCC aggressiveness and metastasis by modulating the EMT process.

### 3.4. COL6A2 Promotes Proliferation, Migration, Invasion, and EMT in ccRCC Cells via the Wnt/β-catenin Signaling Pathway

**Figure 4:**
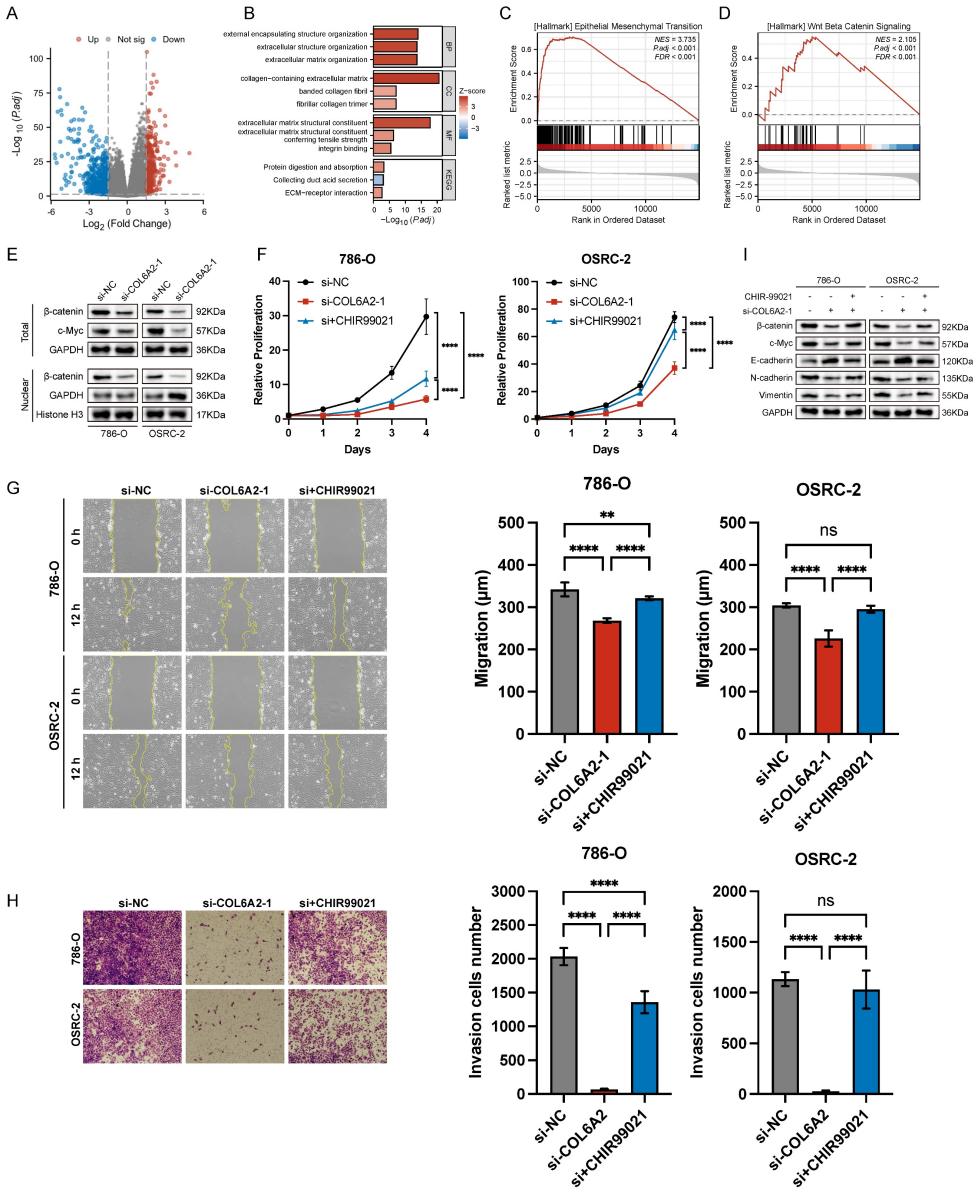
COL6A2 drives ccRCC progression via Wnt/β-catenin signaling. (**A**) Volcano plot of differentially expressed genes (DEGs) between high and low COL6A2 expression groups in the ccRCC TCGA cohort (|logFC| > 1.5 and p.adj < 0.05). (**B**) GO and KEGG enrichment analysis of DEGs. (**C, D**) Gene Set Enrichment Analysis (GSEA) indicating enrichment of EMT and Wnt/β-catenin pathways. (**E**) Western blot showing reduced Wnt/β-catenin pathway protein levels following COL6A2 knockdown in 786-O and OSRC-2 cells. (**F-I**) The Wnt/β-catenin activator CHIR99021 rescues COL6A2 knockdown-induced inhibition of (**F**) proliferation (CCK8), (**G**) migration (wound healing), (**H**) invasion (Transwell), and (**I**) Wnt/β-catenin and EMT protein expression. *p < 0.05, **p < 0.01, ***p < 0.001, ****p < 0.0001; ns, not significant.

A differential gene expression analysis of the TCGA-KIRC cohort was conducted to uncover pathways associated with COL6A2's oncogenic function. By comparing tumors with high versus low COL6A2 expression, and applying significance cutoffs of |LogFC| > 1.5 and p.adj < 0.05, we identified 1073 differentially expressed genes (DEGs) (Fig. 4A). Subsequent KEGG and GO functional enrichment analyses demonstrated a strong association between elevated COL6A2 expression and extracellular matrix (ECM) remodeling (Fig. 4B). Existing evidence suggests that aberrant ECM deposition and structural alterations modify the tumor microenvironment's physicochemical properties, promoting ccRCC progression, invasion, and metastasis 33-35. Furthermore, GSEA (p.adj < 0.05) indicated significant enrichment of gene sets associated with HALLMARK_EPITHELIAL_MESENCHYMAL_TRANSITION and HALLMARK_WNT_BETA_CATENIN_SIGNALING in the high COL6A2 expression group (Fig. 4C, D). These results imply that COL6A2 may drive ccRCC malignancy by modulating the tumor microenvironment to activate Wnt/β-catenin signaling and induce EMT.

To confirm COL6A2's regulatory effect on the Wnt/β-catenin pathway, we evaluated protein expression via Western blot. Knockdown of COL6A2 in 786-O and OSRC-2 cells markedly reduced total β-catenin, nuclear β-catenin levels and c-Myc (Fig. 4E). This indicates that COL6A2 overexpression in ccRCC activates the Wnt/β-catenin signaling pathway.

To definitively establish that COL6A2 drives EMT via the Wnt/β-catenin pathway, we performed a rescue experiment. Specifically, we treated COL6A2-silenced 786-O and OSRC-2 cells with CHIR99021, a pharmacological activator of Wnt/β-catenin signaling, to determine if restoring pathway activity could reverse the effects of the knockdown. CCK-8 and Transwell assays showed that CHIR99021 significantly restored proliferation, migration, and invasion in COL6A2-knockdown cells (Fig. 4F, G, H). Western blot analysis further revealed that, compared to COL6A2 knockdown alone, combined knockdown and CHIR99021 treatment upregulated total β-catenin and c-Myc expression, reduced E-cadherin levels, and increased N-cadherin and vimentin expression (Fig. 4I). These findings demonstrate that activation of the Wnt/β-catenin signaling counteracted the suppressive effects of COL6A2 knockdown on ccRCC cell aggressiveness and EMT.

Collectively, these findings strongly suggest that COL6A2 promotes EMT and enhances the proliferative, migratory, and invasive properties of ccRCC cells, largely through the activation of the Wnt/β-catenin signaling pathway.

### 3.5. COL6A2 engages integrins to modulate Wnt/β-catenin signaling

**Figure 5:**
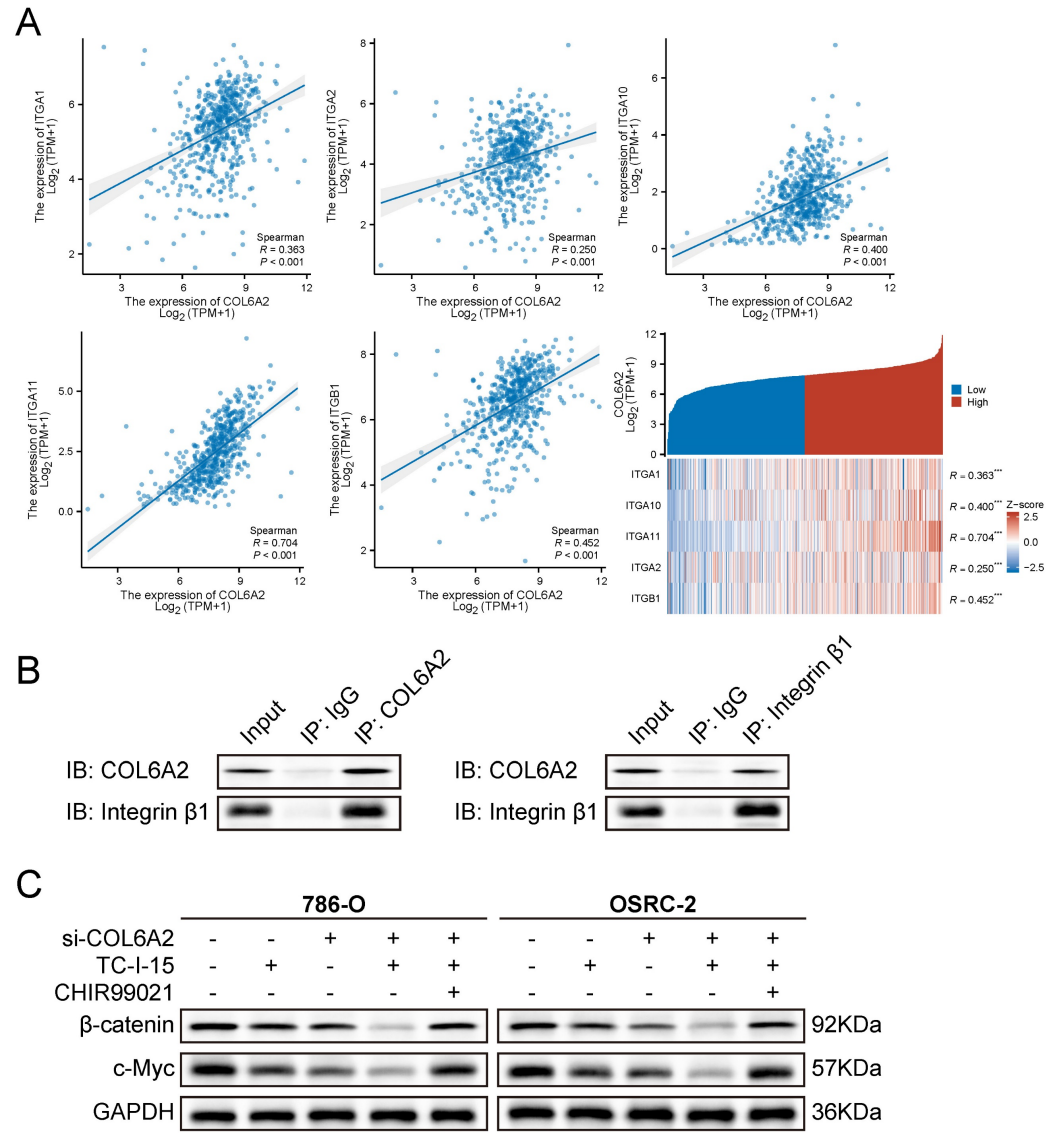
COL6A2 engages integrins to modulate Wnt/β-catenin signaling in ccRCC. (**A**) TCGA-KIRC co-expression analysis shows a significant positive correlation between COL6A2 mRNA and collagen-binding integrin subunits (ITGA1, ITGA2, ITGA10, ITGA11, ITGB1). (**B**) Reciprocal co-immunoprecipitation (co-IP) in OSRC-2 cells demonstrates an association between COL6A2 and integrin β1. COL6A2 immunoprecipitates (IP) contain integrin β1, and integrin β1 IP contain COL6A2; IgG serves as the negative control and Input is shown. (**C**) Western blot analysis of total β-catenin and c-Myc expression in 786-O and OSRC-2 cells. Cells were transfected with si-NC or si-COL6A2 and then treated with the integrin inhibitor TC-I-15 (5 µM, 24 h) and/or the Wnt/β-catenin activator CHIR99021 (10 µM, 24 h) as indicated. *p < 0.05, **p < 0.01, ***p < 0.001, ****p < 0.0001; ns, not significant. Abbreviations: IP, immunoprecipitation; IB/WB, immunoblot.

To elucidate the mechanism by which the extracellular matrix protein COL6A2 activates the intracellular Wnt/β-catenin pathway, we investigated the potential role of integrins as transmembrane mediators. We first performed a co-expression analysis using the TCGA-KIRC dataset, which revealed a significant positive correlation between COL6A2 mRNA levels and the expression of several key collagen-receptor integrin subunits, including ITGA1, ITGA2, ITGA10, ITGA11, and ITGB1 (Fig. 5A). This bioinformatic evidence suggested that COL6A2 may interact with integrin complexes on the surface of ccRCC cells.

To determine if this correlation reflects a physical interaction, we performed reciprocal co-immunoprecipitation (Co-IP) assays in OSRC-2 cells, which exhibit high endogenous COL6A2 expression. The results demonstrated a robust interaction between the two proteins. As shown in Fig. 5B, integrin β1 was successfully detected in the protein complex immunoprecipitated with an anti-COL6A2 antibody. Conversely, COL6A2 was also detected in the complex pulled down by an anti-integrin β1 antibody. Neither protein was detected in the respective IgG control groups. Together, these results provide direct evidence of a physical interaction between COL6A2 and integrin β1 in ccRCC cells.

Finally, we sought to functionally validate the role of this interaction in Wnt/β-catenin signaling. We treated 786-O and OSRC-2 cells with TC-I-15, a pharmacological inhibitor of integrin signaling. Western blot analysis showed that, consistent with COL6A2 knockdown, treatment with TC-I-15 alone significantly reduced the expression of total β-catenin and its downstream target c-Myc (Fig. 5C). Notably, the combined treatment of COL6A2 silencing and integrin inhibition (si-COL6A2 + TC-I-15) resulted in a more profound downregulation of β-catenin and c-Myc compared to either treatment alone. This additive effect suggests that integrin signaling is a major mediator of COL6A2 function, but other mechanisms may also contribute. Furthermore, the suppression of Wnt/β-catenin signaling in the combined treatment group was effectively reversed by the addition of CHIR99021, a Wnt pathway activator (Fig. 5C).

Collectively, these findings demonstrate that COL6A2 physically interacts with integrin β1 and that this engagement is a key mechanism for activating Wnt/β-catenin signaling pathway in ccRCC cells.

## 4. Discussion

The complex interplay within the tumor microenvironment (TME) significantly influences the progression and therapeutic response of ccRCC. Currently, major causes of mortality in advanced ccRCC patients include disease progression driven by drug resistance, as well as, less frequently, fatal immune-related adverse events associated with immune checkpoint inhibitor (ICI) use 12-15. During tumor development, the extracellular matrix (ECM), a critical component of the TME, undergoes substantial remodeling, particularly characterized by aberrant collagen deposition 33-35. The present study, which focused on elucidating the role of COL6A2 in ccRCC, provides compelling evidence of its marked upregulation across both clinical tumor specimens and *in vitro* cell line models. Elevated COL6A2 expression was further correlated with more aggressive clinicopathological characteristics and unfavorable patient prognosis. Mechanistically, we identified that COL6A2 promotes the proliferation, migration, and invasion of ccRCC cells by inducing EMT via modulation of the Wnt/β-catenin signaling in an integrin-dependent manner. Collectively, our findings establish COL6A2 as a potential key pathogenic factor in ccRCC.

Our study demonstrates significant upregulation of COL6A2 in ccRCC. Elevated COL6A2 expression was consistently observed at both the transcript and protein levels across various datasets, including the TCGA-KIRC dataset, GEO datasets (GSE167093 and GSE40435), ccRCC cell lines, and clinical samples from our institution. Moreover, increased COL6A2 expression was positively associated with aggressive clinicopathological features of ccRCC, such as advanced T stage, lymph node metastasis, higher histological grade, and later pathological stage. Kaplan-Meier survival analyses further showed that patients with high COL6A2 expression experienced significantly shorter OS, DSS, and PFI. Importantly, both logistic regression and subsequent Cox proportional hazards regression analyses confirmed high COL6A2 expression as an independent predictor of poor prognosis, highlighting its potential clinical value. The results of our study corroborate the previous findings of Zhong *et al.*, who observed high COL6A2 expression correlated with poor prognosis in metastatic ccRCC, based on 3 additional GEO datasets (GSE85258, GSE105288, and GSE22541) 28. This observation also aligns with previous studies reporting elevated expression of COL6A2 or other type VI collagen chains in various malignancies, including glioma, breast cancer, lung cancer, colorectal cancer, and gastric cancer 20-27. Increased deposition of ECM components, particularly fibrillar collagens and associated proteins, is recognized to contribute to increased tumor stiffness, altered tumor microenvironment, and adverse clinical outcomes in solid tumors 33-36. Collectively, these data strongly support the potential of COL6A2 as a prognostic biomarker in ccRCC.

*In vitro* functional assays demonstrated that silencing of COL6A2 markedly suppressed the proliferation, migration, and invasion of 786-O and OSRC-2, accompanied by decreased expression of proliferation and EMT-related proteins. This molecular shift indicates a reversal of the EMT process. Therefore, elevated COL6A2 expression in ccRCC likely facilitates tumor progression by enhancing cellular proliferation and promoting EMT-mediated invasiveness. These findings align with the oncogenic role of COL6A2 observed in other cancers. For example, Hong *et al.* found that COL6A2 enhances proliferation, motility, invasion, and drug resistance in glioma 20. Similarly, elevated COL6A2 expression has been closely linked to malignant clinical features and poor prognosis in breast cancer, lung adenocarcinoma, and gastric cancer 22, 23, 26. Additionally, EMT-mediated oncogenic effects have also been attributed to COL6A2 in colorectal cancer 24. Collectively, these studies strongly support our observations and indicate that the oncogenic role of COL6A2 across various cancers may involve shared mechanisms, particularly the regulation of EMT.

This study supports that the Wnt/β-catenin pathway is a critical downstream mediator of COL6A2's oncogenic effects in ccRCC. Enrichment analyses, including KEGG, GO, and GSEA, revealed significant concentration of differentially expressed genes (DEGs) in two key areas: pathways governing the EMT and those involved in Wnt/β-catenin signaling. Western blotting experiments confirmed that COL6A2 knockdown significantly reduced total β-catenin, nuclear β-catenin and c-Myc levels, which, along with concurrent changes in EMT markers, indicated suppression of Wnt/β-catenin signaling and reversal of EMT. Furthermore, rescue experiments using the Wnt/β-catenin activator CHIR99021 partially reversed the inhibitory effects of COL6A2 knockdown on both the Wnt/β-catenin pathway and EMT, while also restoring cell proliferation, migration, and invasion. These results strongly support a mediating role for Wnt/β-catenin signaling in COL6A2-induced oncogenesis in ccRCC. The pivotal role of the Wnt/β-catenin pathway in driving EMT and metastasis across various cancers is well-established, where pathway activation typically leads to β-catenin nuclear translocation and subsequent transcriptional activation of EMT-related genes 37. Furthermore, previous research has also linked Collagen VI to Wnt signaling; for instance, Cha *et al.* demonstrated that Collagen VI secreted by glioma cells activates the β-catenin pathway, inducing a mesenchymal phenotype and promoting cell invasion 38. Our study aligns with these findings and extends this mechanism to ccRCC, where COL6A2, an ECM component, promotes EMT at least in part by modulating the Wnt/β-catenin pathway.

Another pivotal advancement of this study is the elucidation of the mechanism that bridges extracellular COL6A2 to the intracellular Wnt/β-catenin pathway. While our initial bioinformatic analyses indicated a strong correlation between COL6A2 expression and Wnt/β-catenin signaling, a direct link was lacking. We hypothesized that integrins, as key cell-surface receptors for ECM proteins, could serve as this missing link 39, 40. Subsequent analysis of the TCGA-KIRC dataset revealed significant positive co-expression between COL6A2 and several collagen-binding integrin subunits. We then provided support for this hypothesis using reciprocal co-immunoprecipitation, which demonstrated an association between COL6A2 and integrin β1 in ccRCC cells. Functionally, pharmacological inhibition of α2β1 (TC-I-15) not only phenocopied the suppressive effects of COL6A2 knockdown on β-catenin and c-Myc but also produced an additive inhibition when combined with COL6A2 silencing; this suppression was rescued by CHIR99021, placing the convergence upstream of GSK3β. Consistent with the well-established role of integrins in “outside-in” signaling, this integrin-dependent mechanism offers a biologically plausible bridge from the tumor microenvironment to oncogenic transcriptional programs in ccRCC 41-43. Notably, the additive suppression observed with combined COL6A2 knockdown and integrin inhibition implies that non-integrin receptors (or co-receptors) may also contribute to COL6A2-mediated Wnt modulation. Candidate mechanisms include other collagen receptors (e.g., discoidin domain receptors) or proteoglycan co-receptors that cooperate with integrins 44, 45. While our current data do not define the precise receptor repertoire or binding interfaces, they motivate future work to map receptor specificity at the subunit level.

Our work sheds new light on the complex molecular pathways that drive ccRCC progression. By mechanistically demonstrating that the ECM protein COL6A2 engages integrin β1 to modulate the Wnt/β-catenin pathway and promote EMT, we have delineated a more complete and actionable signaling cascade. From a translational perspective, our findings identify the COL6A2-integrin interaction as a more precise and therapeutically actionable target than COL6A2 itself. This insight suggests several strategies that merit preclinical evaluation in ccRCC models with high co-expression of COL6A2 and ITGB1. One approach is the direct inhibition of the interaction, either at the receptor level using agents targeting β1 integrins or, more specifically, by disrupting the COL6A2-integrin interface with monoclonal antibodies or decoy peptides once tumor-selective epitopes are defined 46-49. An alternative strategy is to blunt the oncogenic signaling downstream by targeting key mechanotransduction nodes, such as with FAK inhibitors 50-52. To enhance specificity, advanced tumor-restricted delivery platforms—including protease-activated antibodies or ECM-binding drug carriers—could improve on-target exposure while limiting engagement with healthy tissue 53, 54. For any of these approaches, biomarker-guided patient selection and the use of pharmacodynamic readouts, such as β-catenin and EMT markers, will be essential for successful clinical translation. However, the therapeutic potential of targeting this axis must be weighed against the significant risks of off-tumor effects, given the crucial roles of collagens and β1 integrins in hemostasis, wound healing, and tissue homeostasis 55-57. Systemic inhibition could elevate bleeding risks or impair tissue repair. To mitigate these challenges, future clinical strategies should prioritize tumor-localized delivery or activation, employ conservative dosing schedules, and incorporate strict exclusion criteria for patients with bleeding diatheses. Moreover, mixed outcomes with broad integrin inhibitors (e.g., the cilengitide experience) underscore the need for context-specific targeting and rational combinations rather than pan-integrin blockade 47. Further biomarker-guided studies, including early-phase evaluation, are warranted to define on-mechanism efficacy and the therapeutic window.

However, we acknowledge several limitations in the current study. Our functional experiments were primarily conducted in *in vitro* cell line models and did not extend to the more complex *in vivo* microenvironment. To address this, we plan to establish xenograft models in future studies to further validate COL6A2's role in ccRCC progression. Furthermore, while our findings support the COL6A2-integrin β1 axis as a major integrin-dependent component of Wnt/β-catenin modulation, the additive effects observed in our blockade experiments suggest that COL6A2 may also operate through other parallel pathways, a possibility that warrants further investigation. Finally, the clinical analysis was restricted by a limited sample size (only 10 ccRCC specimens), and due to insufficient follow-up time, clinical outcome data for these specific patients were not collected. Consequently, further validation of the clinical utility and prognostic significance of COL6A2 in larger, independent, and prospectively collected patient cohorts is warranted.

## 5. Conclusion

In summary, our data support an oncogenic role for COL6A2 in ccRCC and indicate that COL6A2 modulates Wnt/β-catenin signaling in an integrin-dependent manner. While additional receptors may contribute, the findings position β1-containing integrins as a mechanistic bridge between extracellular COL6A2 and intracellular signaling. These insights highlight the COL6A2-integrin axis as a potential biomarker and therapeutic target in ccRCC and provide a rationale for strategies that disrupt ECM-integrin interactions to temper Wnt/β-catenin activity.

## Figures and Tables

**Table 1:** Logistic analysis of COL6A2 and clinical characteristics in ccRCC.

Characteristics	Total (N)	OR (95% CI)	P value
Pathologic T stage (T4 vs. T1&T2&T3)	541	2.901 (1.691 - 4.978)	< 0.001
Pathologic N stage (N1 vs. N0)	258	1.574 (1.008 - 2.458)	0.046
Pathologic M stage (M1 vs. M0)	508	1.257 (1.017 - 1.555)	0.035
Pathologic stage (Stage III&IV vs. Stage I&II)	538	1.294 (1.110 - 1.508)	< 0.001
Histologic grade (G4 vs. G1&G2&G3)	533	1.838 (1.439 - 2.349)	< 0.001
Age (> 60 vs. <= 60)	541	0.882 (0.767 - 1.015)	0.079
Gender (Female vs. Male)	541	0.974 (0.843 - 1.125)	0.716

**Table 2:** Cox regression analysis of variables for OS in ccRCC.

Characteristics	Total(N)	Univariate analysis		Multivariate analysis	
		Hazard ratio (95% CI)	P value	Hazard ratio (95% CI)	P value
Pathologic T stage	541				
T1&T2&T3	530	Reference		Reference	
T4	11	5.943 (3.127 - 11.293)	**< 0.001**	1.361 (0.451 - 4.111)	0.585
Pathologic N stage	258				
N0	242	Reference		Reference	
N1	16	3.422 (1.817 - 6.446)	**< 0.001**	1.425 (0.577 - 3.518)	0.443
Pathologic M stage	508				
M0	429	Reference		Reference	
M1	79	4.401 (3.226 - 6.002)	**< 0.001**	2.410 (1.412 - 4.111)	**0.001**
Pathologic stage	538				
Stage I& Stage II	332	Reference		Reference	
Stage III& Stage IV	206	3.910 (2.852 - 5.360)	**< 0.001**	1.691 (0.985 - 2.902)	0.057
Histologic grade	533				
G1&G2	250	Reference		Reference	
G3&G4	283	2.665 (1.898 - 3.743)	**< 0.001**	1.663 (1.014 - 2.729)	**0.044**
Age	541				
<= 60	269	Reference		Reference	
> 60	272	1.791 (1.319 - 2.432)	**< 0.001**	1.810 (1.173 - 2.793)	**0.007**
Gender	541				
Male	354	Reference			
Female	187	1.083 (0.796 - 1.473)	0.613		
COL6A2	541	1.293 (1.127 - 1.483)	**< 0.001**	1.354 (1.108 - 1.656)	**0.003**
